# The role of serum and urinary urea in the evaluation of enteral protein intake in adequate and small-for-gestational-age very low birth weight infants

**DOI:** 10.1590/S1516-31802005000600002

**Published:** 2005-11-01

**Authors:** Silvana Darcie, Cléa Rodrigues Leone

**Keywords:** Gestational age, Small for gestational age infant, Very low birth weight infant, Urea, Idade gestacional, Recém-nascido pequeno para a idade gestacional, Recém-nascido de muito baixo peso, Uréia

## Abstract

**CONTEXT AND OBJECTIVE:**

Very low birth weight (VLBW) infants have special nutritional needs. There is a current tendency to individualize their protein needs. The objective of this study was to determine the suitability of serum and urinary urea as indicators for protein intake in adequate-for-gestational-age (AGA) and small-for-gestational-age (SGA) VLBW infants.

**DESIGN AND SETTING:**

Prospective study in the nursery attached to the Maternity Ward of the “Prof. Pedro de Alcântara” Children's Institute, Hospital das Clínicas, Department of Pediatrics, Faculdade de Medicina da Universidade de São Paulo, Brazil.

**METHODS:**

Seventy-two VLBW infants (mean protein intake = 3.7 mg/kg/day) were enrolled in a prospective cohort study in two groups: AGA (n = 34) and SGA (n = 38). Blood samples, six-hour urine (6hUr) collections and urine sample tests (STUr) were obtained for urea and creatinine assays at three and five weeks of life. Statistical analysis: Student's t test, Pearson correlation and linear regression (p < 0.05).

**RESULTS:**

There were no differences between groups for serum urea, 6hUr and STUr, or between two assessments within each group. Serum urea correlated with 6hUr in both AGA and SGA, and to STUr in SGA; 6hUr correlated with STUr in both AGA and SGA. There was no correlation between protein intake and serum or urine urea.

**CONCLUSIONS:**

Serum and urinary urea did not reflect protein intake when mean intakes of 3.7 g/kg/day were used. Sample tests of urinary urea can be as reliable as urea from urine collected over longer periods.

## RESUMO

**INTRODUÇÃO E OBJETIVOS:**

Os recém-nascidos de muito baixo-peso (RNMBP) têm necessidades nutricionais especiais. Existe uma tendência atual de se individualizar a oferta protéica para essas crianças. O objetivo do trabalho é determinar a utilidade da uréia sérica e urinária como indicadores da oferta protéica em RNMBP adequados (AIG) e pequenos para a idade gestacional (PIG).

**TIPO DE ESTUDO E LOCAL:**

Estudo prospectivo realizado no Berçário Anexo à Maternidade Instituto da Criança “Prof. Pedro de Alcântara” do Hospital das Clínicas, Departamento de Pediatria da Faculdade de Medicina, Universidade de São Paulo, Brasil.

**MÉTODOS:**

Setenta e dois RNMBP (oferta protéica média = 3,7 g/kg/dia) foram incluídos, em um estudo de coorte prospectivo, em dois grupos: AIG (n = 34) e PIG (n = 38). Amostras de sangue, coletas de urina de seis horas (Ur6h) e em amostras isoladas (AIUr) foram obtidas para determinação de uréia e creatinina após a 3^a^ semana de vida e duas semanas após. Análise estatística: teste t de Student, correlação de Pearson e regressão linear (p < 0,05).

**RESULTADOS:**

Não houve diferença entre os grupos quanto aos níveis de uréia sérica, uréia Ur6h e uréia AIUr, bem como entre as duas avaliações dentro de cada grupo. A uréia sérica correlacionou-se à uréia Ur6h nos RNAIG e nos PIG, bem como à uréia AIUr nos RNPIG. A uréia Ur6h correlacionou-se à uréia AIUr nos RNAIG e nos RNPIG. Não houve correlação entre a oferta protéica e a uréia sérica ou urinária.

**CONCLUSÕES:**

A uréia sérica e a urinária não refletiram a oferta protéica quando foram utilizadas ofertas médias de 3,7 g/kg/dia. Uréia AIUr pode ser tão confiável quanto uréia da urina coletada por períodos mais longos.

## INTRODUCTION

Very low birth weight (VLBW) infants have special nutritional needs due to their fast growth rates and higher incidence of clinical complications.^1–3^ Therefore, they require higher amounts of protein per unit body weight than do term infants.^4^ However, they are vulnerable to protein metabolism imbalances because of their metabolic immaturity.^5–7^ As a consequence, it is necessary to individualize their protein needs by means of indicators to assess the adequacy of protein intake for these infants.

Many methods can be used to assess the adequacy of protein intake: anthropometric measurements, biochemical parameters, serum amino acid profile, isotopic methods, nutritional and energy balances. However, such methods need to be reliable, non-invasive, non-time consuming and economical. Among them, serum and urinary urea may be simple and economical methods, since urea can be considered to be an important way of adjusting nitrogen excretion in relation to protein intake.^8–10^ Moreover, a previous study found good correlation between protein intake and serum or eight-hour urinary urea in very low birth weight infants.^6^

## OBJECTIVE

On the basis of the previous findings, a prospective cohort study was performed with the objective of testing the hypothesis that serum and urinary urea may be simple and inexpensive methods for assessing the adequacy of protein intake for adequate-for-gestational-age (AGA) and small-for-gestational-age (SGA) VLBW infants. It was also assessed whether sample tests of urinary urea would be as reliable as urine collected over longer periods.

## MATERIALS AND METHODS

Seventy-two VLBW infants from the nursery attached to the Maternity Ward of the “Prof. Pedro de Alcântara” Children's Institute, Hospital das Clínicas, Department of Pediatrics, Faculdade de Medicina da Universidade de São Paulo, which is a tertiary university hospital, were enrolled over a 16-month period, from April 7, 1997 up to August 21, 1998. They were all healthy preterm infants (with less than 36 weeks of gestational age and less than 1,500 g) with postnatal age of more than three weeks. None of them presented malformations, none were on mechanical ventilation, none presented infection, and they were exclusively receiving their own mother's milk ³ 120 ml/kg/day and/or preterm formula via enteral route.

The gestational age was determined from the date of the last normal menstrual period, if compatible with the date determined by ultrasonography (US), and it was verified by clinical evaluation using the New Ballard method.^11^ When the clinical assessment differed from the calculated gestational age (using the last normal menstrual period/US) by two weeks or more, the definitive gestational age was based on clinical assessment. Infants were classified as AGA if their birth weights were above the 10^th^ percentile and below the 90^th^ percentile of the Ramos Intrauterine Growth Curve,^12^ and as SGA if their birth weights were below the 10^th^ percentile of the same curve. The infants were then divided into two groups: AGA (n = 34) and SGA (n = 38).

Informed consent was obtained from the mothers in all cases and the study protocol had previously been approved by the local ethical committee.

Blood samples, six-hour urine collections and sample tests of urine were obtained at two times, for urea and creatinine assays: the first assessment was performed when the newborn was three weeks old or more, and the second one was performed two weeks later, if the infant was still hospitalized. Blood sampling was preprandial, during the six-hour urine collection, and the samples were immediately sent to the laboratory or centrifuged, separated and frozen. The six-hour and sample tests of urine were collected in plastic bags. The urine samples were frozen until analyzed. The blood and urine were analyzed for urea using the Ultraviolet Enzyme Kinetic Test and for creatinine using Jaffé's buffered kinetic reaction without deproteinization.^13^ Creatinine clearance was calculated by the usual formula: creatinine clearance (ml/min/1.73 m^2^) = (UV/S) x (1.73 / BS)m^2^, where U = urinary creatinine concentration (mg/dl), V = urinary volume divided by the duration of the collection period in minutes, S = serum creatinine concentration (mg/dl) and BS = body surface (m^2^) calculated by the formula (4 x body weight) + 7/body weight + 90. The mean calorie-protein intake was calculated for each assessment on the basis of feed volumes (obtained from nursing records) and the calorie-protein content of the milk: mother's milk = 67 kcal/100 ml and 1.2 g/100 ml,^7^ respectively, or preterm formula (PreNan^®^, Nestlé) = 70 kcal/100 ml and 2.0 g/100 ml at 14.3% dilution, respectively, and 80 kcal/100 ml and 2.35 g/100 ml at 16.3% dilution, respectively, according to the manufacturer's instructions.

Anthropometric measurements were performed at the first assessment, one week later and at the second assessment of blood samples. The newborn was weighted on an electronic balance (Filizola, São Paulo, Brazil) to the nearest five grams. Crown-heel length was measured by using a wooden measuring board with a fixed headboard and a movable footboard (cm). Head circumference (largest occipital-frontal circumference) was measured using a glass fiber measuring tape (cm).

### Data analysis

Data were entered into the Sigma Stat^®^ program for analysis, and the Sigma Plot^®^ program was used to create graphics.

The anthropometric and demographic variables were compared between groups by means of Student's t-test and Fisher's exact test. Correlations between serum and the six-hour and sample tests of urinary urea, and between the last two, were made using Pearson's correlation. Linear regression models for serum and the six-hour and sample tests of urinary urea were adjusted by considering protein intake and creatinine clearance as independent variables. The significance level was set at p < 0.05.

## RESULTS

There were 2,785 infants born at the nursery attached to the Maternity Ward during the study period; 144 of them (5.2%) were VLBW infants. Forty-seven died and 25 were excluded because they were not exclusively receiving enteral feeding. Seventy-two (74.2%) were included in this study.

Thirty-four of the 72 newborns included were assessed twice (first and second assessments): 15 in group I and 19 in group II. These infants were still hospitalized two weeks after the first assessment (Table 1).

**Table 1.** Characteristics of 72 very low birth weight newborns in a tertiary university hospital in São Paulo, Brazil

	Total	AGA Group	SGA Group	p
n	72	34	38	-
Mean birth weight (g)	1259.5	1343.8	1184.1	< 0.001
(SD)	(182.0)	(105.3)	(203.2)	
Mean gestational age (weeks)	31.6	30.8	32.3	< 0.001
(SD)	(2.0)	(1.1)	(2.3)	
Male (%)	45.8	61.8	31.6	0.017
White (%)	55.6	52.9	57.9	0.813
Mean postnatal age (days)	-	23.2	24.3	0.119
(SD)		(2.6)	(2.9)	
First assessment				
Mean postnatal age (days)		36.3	37.8	0.167
(SD)		(3.0)	(3.4)	
Second assessment				
Mean PCA (weeks)	-	34.1	35.8	< 0.001
(DP)		(1.3)	(2.4)	
First assessment				
Mean PCA (weeks)	-	35.3	36.7	0.031
(DP)		(1.2)	(2.1)	
Second assessment				
Newborns with 2 assessments	34	15	19	

*AGA Group = adequate-for-gestational-age very low birth weight infants; SGA Group = small-for-gestational-age very low birth weight infants; PCA = postconceptional age; SD = standard deviation.*

When it was necessary to compare results between the first and second assessments within each group, only the newborns assessed twice were taken into account. This procedure was used in order to avoid bias, because the newborns assessed only once might have been those with a more favorable outcome and, consequently, faster discharge.

The mean gestational age was 31.6 ± 2.0 weeks (range: 26.1-35.7 weeks) and the mean birth weight was 1259.5 ± 182.0 g (range: 730-1490 g). Thirty-nine (54.2%) were female and 33 (45.8%) were male. Forty (55.6%) were white and 32 (44.4%) were non-white. These characteristics as well as group-specific characteristics are presented in Table 1.

Both groups showed adequate weight and height gain, and also adequate increase in head circumference.

The mean protein and energy intake and the mean creatinine clearance for both groups are showed in Table 2. Only three infants in each group were fed using mother's milk.

**Table 2.** Mean protein (g/kg/day) and energy (kcal/kg/day) intake and creatinine clearance (ml/min/1.73 m^2^) at each assessment, for both groups of very low birth weight infants (n = 72) studied in a Brazilian tertiary university hospital

	AGA Group	SGA Group	^p^
Protein intake – first assessment	3.7	3.8	0.163
(SD)	(0.5)	(0.6)	
n	34	38	
Protein intake – second assessment	3.9	3.9	0.655
(SD)	(0.4)	(0.5)	
n	15	19	
Energy intake – first assessment	127.3	134.0	0.032
(SD)	(12.3)	(13.6)	
n	34	38	
Energy intake – second assessment	133.5	135.1	0.753
(SD)	(13.4)	(15.8)	
n	15	19	
Creatinine clearance – first assessment	18.1	20.1	0.418
(SD)	(7.0)	(12.9)	
n	32	38	
Creatinine clearance – second assessment	33.2	23.2	0.033
(SD)	(13.8)	(11.0)	
n	15	16	

*AGA Group = adequate-for-gestational-age very low birth weight infants; SGA Group = small-for-gestational-age very low birth weight infants; SD = standard deviation.*

**Figure 1:**
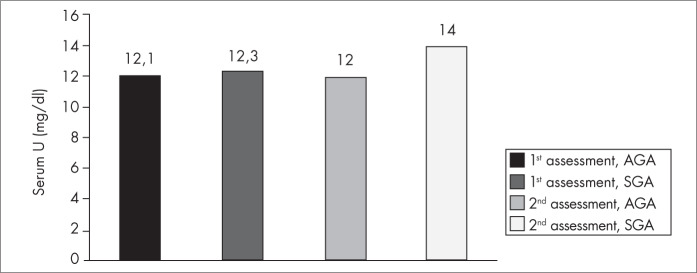
Serum urea concentrations (mg/dl) at the first and second assessments, for both groups of very low birth weight infants (total of 72 infants): adequate for gestational age (AGA) and small for gestational age (SGA).

**Figure 2:**
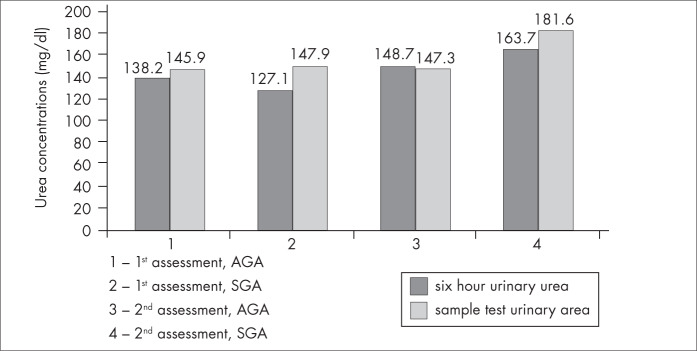
Six-hour and sample test urinary urea concentrations (mg/dl) at the first and second assessments, for both groups of very low birth weight infants (total of 72 infants): adequate for gestational age (AGA) and small for gestational age (SGA).

There were no differences in serum urea, urea from six-hour urine collections and sample tests of urinary urea. Likewise, there were no differences between the two assessments within each group (Figures 1 and 2).

**Figure 3:**
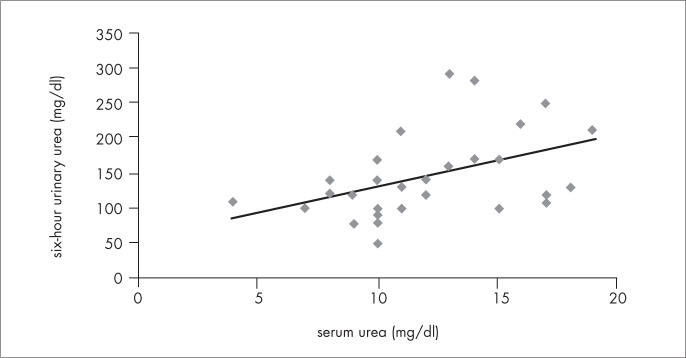
Correlation between serum and six-hour urinary urea in adequate-for-gestational-age (AGA) very low birth weight infants at the first assessment (r = 0.439; p = 0.017), performed when infants were three weeks old.

**Figure 4:**
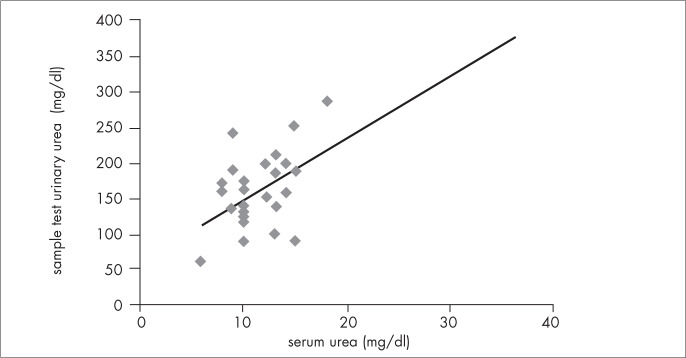
Correlation between serum and sample test urinary urea for small-forgestation-age (SGA) very low birth weight infants at the first assessment (r=0.731; p<0.001), performed when infants were three weeks old.

**Figure 5:**
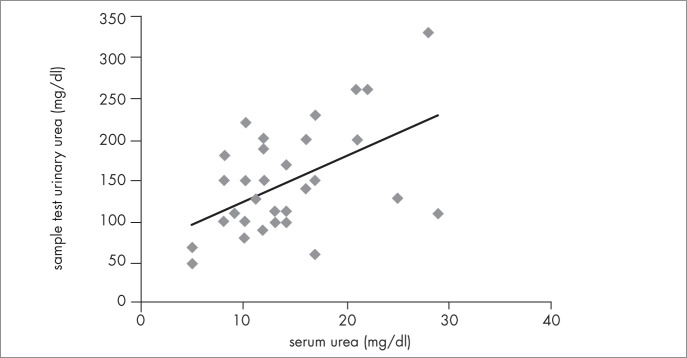
Correlation between six-hour and sample test urinary urea for adequate-forgestational age (AGA) very low birth weight infants at the first assessment (r = 0.515; p < 0.01), performed when infants were three weeks old.

Serum urea correlated with six-hour urinary urea in both AGA (first assessment: r = 0.44; p = 0.02; second assessment: r = 0.82; p < 0.001) and SGA (first: r = 0.67; p < 0.001; second: r = 0.63; p = 0.03), as shown in Figure 3, and also with sample tests of urinary urea in SGA (first: r = 0.73; p < 0.001; second: r = 0.75; p < 0.01) (Figure 4). There was a trend towards a correlation between serum urea and sample tests of urinary urea in AGA (first: r = 0.29; p = 0.131; second: r = 0.46; p = 0.11). Six-hour urinary urea correlated with sample tests of urinary urea in both AGA (first: r = 0.52; p < 0.01; second: r = 0.71; p < 0.01) and SGA (first: r = 0.62; p < 0.001; second: r = 0.76; p < 0.001) (Figure 5).

There was no correlation between protein intake and serum or urinary urea (six-hour and sample test), even taking creatinine clearance into account, in either group (Table 3).

**Table 3.** Correlation of protein intake and creatinine clearance with serum urea, six- hour urinary urea and sample test urinary urea at the first (when newborn was three weeks old or more) and second (two weeks later) assessments, for both groups of very low birth weight newborns: adequate or small for gestational age

		AGA Group		SGA Group	
Assessment		First	Second	First	Second
Protein intake	^p^	0.440	0.550	0.563	0.411
X					
Creatinine clearance	p	0.244	0.086	0.722	0.441
X					
Serum urea	n	28	13	25	12
Protein intake	p	0.771	0.163	0.169	0.409
X					
Creatinine clearance	p	0.343	0.031	0.640	0.773
X					
Six-hour urinary urea	n	32	15	38	16
Protein intake	p	0.079	0.279	0.934	0.387
X					
Creatinine clearance	p	0.910	0.466	0.872	0.935
X					
Sample test urinary urea	n	32	15	38	16

*AGA Group = adequate-for-gestational-age very low birth weight infants; SGA Group = small-for-gestational-age very low birth weight infants.*

## DISCUSSION

There is a current tendency towards individualizing VLBW infants’ protein needs, in spite of the various protein intake recommendations for them in the medical literature. Different methods can be used to assess the adequacy of enteral protein intake. Among these, serum and urinary urea may be simple, non-invasive, non-time consuming and economical methods, particularly sample tests of urinary urea.^14^

We did not find studies in the medical literature that used sample tests of urinary urea to evaluate the adequacy of protein intake for VLBW infants. Our data showed a positive correlation between sample tests of urinary urea and six-hour urinary urea in AGA and SGA, not only in the first assessment, but also in the second one. There was also good correlation between sample tests of urinary urea and serum urea in the two groups, at both assessments (Figure 4).

It is important to emphasize that creati-nine clearance did not explain the variations in urea levels in the sample tests of urinary urea when the multiple linear regression models were adjusted, taking serum and urinary urea as the dependent variables and creatinine clearance as the independent variable.

These results open up the prospect of using urine sample tests to assess urea concentrations, thereby doing away with the need for longer periods of urine collection.

Polberger et al.^6^ analyzed urea concentrations in serum and eight-hour urine collections in 28 growing, appropriate-for-gestational-age VLBW infants that were fed with a range of human milk protein intakes, from 1.7 to 3.9 g/kg/day. They found a high correlation between serum urea values and mean protein intake, and between urinary urea concentrations in eight-hour urine collections and protein intake. However, we did not find any correlation between serum or urea (six-hour or sample test urea) and protein intake.

In the study by Polberger et al.,^6^ the correlation between serum or eight-hour urine collections and protein intake was examined by feeding two groups of AGA VLBW infants with diets of different energy content (113.3 ± 9.5 and 126.8 ± 12.0 kcal/kg/day, respectively) and protein (2.12 0,30 and 3.59 0.25 g/kg/day) content. The mean birth weight (1,240 ± 175 and 1,200 145 g, respectively) and mean gestational age (28.5 ± 1.0 and 28.6 ± 1.5 weeks, respectively) were similar to those of the infants included in the present study (Table 1). Also, the postnatal ages at which the assessments were performed (27.9 5.8 and 28.3 7.6 weeks, respectively) were similar to those in this study (Table 1). As can be seen in their graphs, they^6^ found a correlation between protein intake and serum or urinary urea only at lower levels of protein intake, i.e. between 2.0 and 2.5 g/kg/day. On the other hand, there was a spread in the points at higher level of protein intake, i.e. between 3.5 and 4.0 g/kg/day, thus indicating a lack of correlation that is similar to what was found in the present study (Table 2). Boehm et al.^15^ also found a correlation between lower protein intakes and serum urea.

It is possible that serum and urinary urea may only correlate with lower protein intakes. This may be attributable to the fact that serum or urinary urea comes from the oxidation of amino acids that results from protein synthesis and degradation balance, i.e. it does not represent only a measurement of new tissue protein accretion.

According to this hypothesis, during lower protein intake, a reduction in amino acid oxidation occurs in order to preserve proteins for amino acid synthesis, which is the main process. As a result, there is a linear relationship between protein intake and urea production. At this point, urea concentrations probably reflect protein synthesis that is the resultant from the utilization of amino acid coming from the diet. On the other hand, during higher protein intake, excess amino acid is oxidized, leading to greater urea production and, as a result, the linear relationship between protein intake and urea disappears. At this point, urea levels reflect not only the synthesis resulting from protein intake, but also the oxidation of amino acid that exceeds the amount needed for the synthesis.

Few studies have addressed the differences in protein metabolism between AGA and SGA VLBW infants.^16–18^ According to some authors,^16^ the nutritional needs for catch-up growth in SGA infants are higher than in AGA infants. However, it is still unknown whether SGA infants are able to metabolize higher protein intakes.

Our results showed no significant difference in mean serum and urinary urea between AGA and SGA infants in either assessment. These data are in accordance with the absence of difference in protein intakes between the two groups in this study. Moreover, there was no difference in serum and urinary urea between the two assessments. This finding could be a consequence of using the same protein intake over the study period.

Some authors^18^ believe that postconceptional age is the most important factor that affects protein metabolism. However, the similar results obtained in the two study groups, with significantly different gestational ages, with regard to serum and urinary urea concentrations, indicate that postconceptional age has a non-significant influence on protein metabolism after the third week of age.

Future research, based on varying the protein intakes and analyzing the influence of other factors on urea concentrations in urine sample tests, will provide better information regarding the role of urea as an indicator for protein intake adequacy.

## CONCLUSIONS

Serum and urinary urea did not reflect protein intake when mean intakes of 3.7 g/kg/day were used. Sample tests of urinary urea can be as reliable as urea from urine collected over longer periods.
